# Three CoA Transferases Involved in the Production of Short Chain Fatty Acids in *Porphyromonas gingivalis*

**DOI:** 10.3389/fmicb.2016.01146

**Published:** 2016-07-19

**Authors:** Mitsunari Sato, Yasuo Yoshida, Keiji Nagano, Yoshiaki Hasegawa, Jun Takebe, Fuminobu Yoshimura

**Affiliations:** ^1^Department of Microbiology, School of Dentistry, Aichi Gakuin UniversityNagoya, Japan; ^2^Department of Removable Prosthodontics, School of Dentistry, Aichi Gakuin UniversityNagoya, Japan

**Keywords:** *Porphyromonas gingivalis*, butyrate, CoA transferase, short chain fatty acid, propionate

## Abstract

Butyryl-CoA:acetate CoA transferase, which produces butyrate and acetyl-CoA from butyryl-CoA and acetate, is responsible for the final step of butyrate production in bacteria. This study demonstrates that in the periodontopathogenic bacterium *Porphyromonas gingivalis* this reaction is not catalyzed by PGN_1171, previously annotated as butyryl-CoA:acetate CoA transferase, but by three distinct CoA transferases, PGN_0725, PGN_1341, and PGN_1888. Gas chromatography/mass spectrometry (GC-MS) and spectrophotometric analyses were performed using crude enzyme extracts from deletion mutant strains and purified recombinant proteins. The experiments revealed that, in the presence of acetate, PGN_0725 preferentially utilized butyryl-CoA rather than propionyl-CoA. By contrast, this preference was reversed in PGN_1888. The only butyryl-CoA:acetate CoA transferase activity was observed in PGN_1341. Double reciprocal plots revealed that all the reactions catalyzed by these enzymes follow a ternary-complex mechanism, in contrast to previously characterized CoA transferases. GC-MS analysis to determine the concentrations of short chain fatty acids (SCFAs) in culture supernatants of *P. gingivalis* wild type and mutant strains revealed that PGN_0725 and PGN_1888 play a major role in the production of butyrate and propionate, respectively. Interestingly, a triple deletion mutant lacking PGN_0725, PGN_1341, and PGN_1888 produced low levels of SCFAs, suggesting that the microorganism contains CoA transferase(s) in addition to these three enzymes. Growth rates of the mutant strains were mostly slower than that of the wild type, indicating that many carbon compounds produced in the SCFA synthesis appear to be important for the biological activity of this microorganism.

## Introduction

Periodontal diseases are a group of inflammatory conditions that lead to the destruction of tooth-supporting tissues and appear to be associated with serious systemic conditions (Kumar, 2013). Among the periodontitis-associated bacteria, *Porphyromonas gingivalis* is the best-studied periodontal pathogen.

*Porphyromonas gingivalis*, which is a Gram-negative, black-pigmented, asaccharolytic anaerobe, is implicated in the initiation and progression of periodontal diseases (Lamont and Jenkinson, 2000). Its primary niche is the anaerobic environment of the sub-gingival pockets (Lamont and Jenkinson, 1998; Yoshida et al., 2003). This organism produces a variety of potential virulence factors, such as fimbriae, hemagglutinins, lipopolysaccharide, capsular polysaccharide, vesicles, outer membrane proteins, and proteolytic enzymes (Holt et al., 1999; Lamont and Jenkinson, 2000; How et al., 2016). *P. gingivalis* also releases large amounts of butyrate and propionate into its culture medium (Niederman et al., 1996; Imai et al., 2012). These molecules easily penetrate the periodontal tissue because of their low molecular weights (Tonetti et al., 1987) and subsequently disturb host cell activity and host defense systems (Singer and Buckner, 1981; Eftimiadi et al., 1990; Kurita-Ochiai et al., 1995). Concentrations of these molecules in the periodontal pockets significantly correlate with the clinical measure of disease severity and inflammation (Niederman et al., 1997; Qiqiang et al., 2012). Furthermore, butyrate, which induces apoptosis in gingival fibroblasts and in T- and B-cells (Kurita-Ochiai et al., 1995, 2000, 2008; Chang et al., 2013), is the most toxic metabolic end product found in the oral cavity (Niederman et al., 1997). In the gastrointestinal tract, however, butyrate produced by bacteria is thought to play an important and beneficial role (Siavoshian et al., 2000; Peng et al., 2009; Plöger et al., 2012; Qin et al., 2012; Le Chatelier et al., 2013; Mathewson et al., 2016).

Two different pathways for the synthesis of butyrate from butyryl-CoA have been characterized to date. The first pathway involves phosphotransbutyrylase and butyrate kinase, with butyryl-CoA converted to butyrate through the formation of a butyryl phosphate intermediate. This pathway was identified in *Clostridium acetobutylicum* (Walter et al., 1993). In the second pathway, butyryl-CoA:acetate CoA transferase transfers the CoA moiety from butyryl-CoA to an exogenous acetate molecule, resulting in the formation of acetyl-CoA and butyrate (Duncan et al., 2002). A screen of butyrate-producing isolates from the human gut suggested that the latter pathway is more prevalent than the former (Louis et al., 2004). A biochemical study using crude enzyme extracts suggested that the latter pathway is also operational in *P. gingivalis* (Takahashi et al., 2000). PGN_1171 was annotated as the CoA transferase associated with the last step of butyrate production in *P. gingivalis* ATCC 33277 (Nelson et al., 2003; Hendrickson et al., 2009).

**FIGURE 1:**
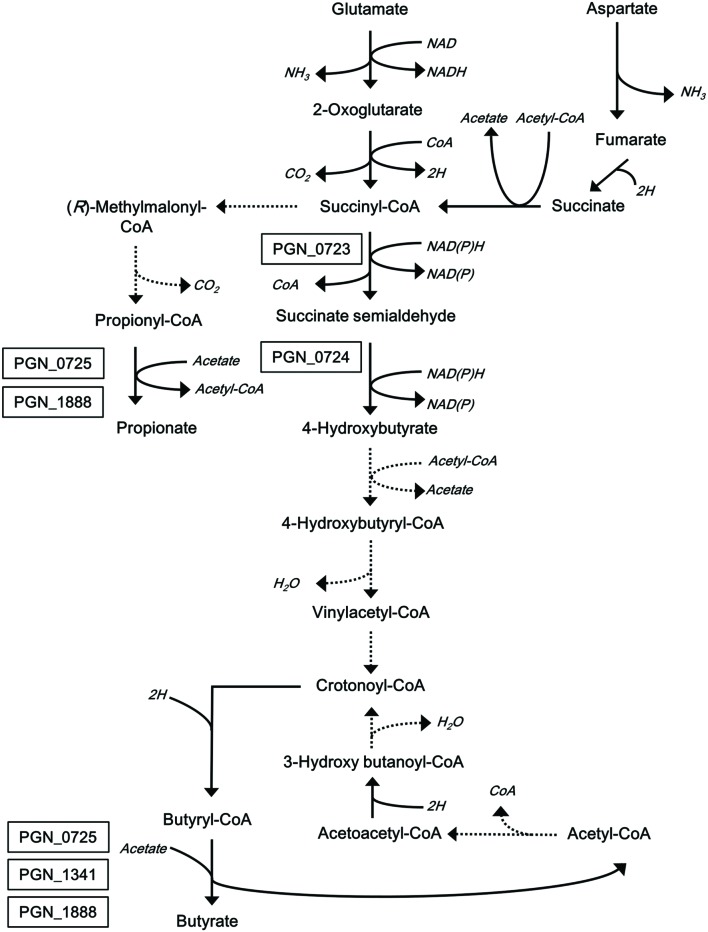
**Proposed metabolic pathways for glutamate and aspartate catabolism in *P. gingivalis***. This scheme was constructed based on previous reports (Takahashi et al., 2000; Hendrickson et al., 2009). Broken lines indicate predicted pathways that have not been supported by experimental data. Associations of the boxed proteins with the pathways have been determined experimentally (Yoshida et al., 2015, 2016).

We recently reported the identification and characterization of two reductases that produce succinate semialdehyde and 4-hydroxybutyrate, both of which are intermediates of the butyrate synthetic pathway of *P. gingivalis* (Yoshida et al., 2015, 2016). We are now extending molecular studies of the butyrate production pathway to the final step of the pathway (**Figure 1**). In this study, we first demonstrate that PGN_1171 is not involved in the reaction of butyrate production from butyryl-CoA, and, instead, we identify three candidate CoA transferases using a homology search with CoA transferase in *Roseburia hominis*, which is an anaerobic intestinal bacterium (Charrier et al., 2006; Travis et al., 2015). To understand their roles in butyrate production in *P. gingivalis*, several deletion strains were constructed, and short chain fatty acids (SCFAs) in their supernatants were quantified and analyzed. In addition, the genes encoding the putative enzymes were cloned and expressed in *Escherichia coli*, and the recombinant proteins were purified and then enzymatically characterized.

## Materials and Methods

### Bacterial Strains and Culture Conditions

*Porphyromonas gingivalis* strains used in this study are listed in **Table 1**, and were grown anaerobically at 37°C in a modified GAM broth (Nissui, Tokyo, Japan) or on Brucella HK agar plates (Kyokuto Pharmaceutical Industrial, Tokyo, Japan), supplemented with 5% rabbit blood. The following antibiotic concentrations were used, as appropriate: 20 μg/ml erythromycin, 0.5 μg/ml tetracycline, and/or 10 μg/ml ampicillin. *E. coli* DH5α and BL21 (DE3) strains were grown aerobically at 37°C in 2× YT medium (Becton Dickinson Japan, Tokyo, Japan) with 100 μg/ml ampicillin, 200 μg/ml erythromycin, or 10 μg/ml tetracycline.

**Table 1:** *Porphyromonas gingivalis* strains and plasmids used in this study.

Strain and plasmid	Relevant characteristics	Reference
***P. gingivalis***		
ATCC 33277	Wild type	ATCC
PGAGU101	*P. gingivalis* ATCC 33277 containing the *erm* cassette in place of PGN_1171	This study
PGAGU104	*P. gingivalis* ATCC 33277 containing the *erm* cassette in place of PGN_0725	This study
PGAGU108	*P. gingivalis* ATCC 33277 containing the *erm* cassette in place of PGN_1888	This study
PGAGU109	*P. gingivalis* ATCC 33277 containing the *erm* cassette in place of PGN_1341	This study
PGAGU111	*P. gingivalis* PGAGU104 containing *tetQ* in place of PGN_1888	This study
PGAGU114	*P. gingivalis* PGAGU109 containing *tetQ* in place of PGN_1888	This study
PGAGU115	*P. gingivalis* PGAGU109 containing *tetQ* in place of PGN_0725	This study
PGAGU118	*P. gingivalis* PGAGU115 containing *cepA* in place of PGN_1888	This study
**Plasmids**		
pVA2198	Em^R^ in *E. coli* and *P. gingivalis*	Fletcher et al., 1997
pT-COW	Ap^R^ and Tc^R^ in *E. coli*, Tc^R^ in *P. gingivalis*	Gardner et al., 1996
pCEPA	Ap^R^ in *E. coli* and *P. gingivalis*; pCR-Blunt II TOPO (Thermo Fisher Scientific) containing *cepA* with its native promoter and terminator	Dr. Naito, Nagasaki University, Japan
pUC19	Ap^R^, vector for cloning	GE Healthcare
pGN1171	Cm^R^ and Em^R^, pUC19 derivative containing the *erm* cassette flanked by upstream and downstream regions of PGN_1171	This study
pKO0725-*erm*	Ap^R^ and Em^R^, pUC19 derivative containing the *erm* cassette flanked by upstream and downstream regions of PGN_0725	This study
pKO0725-*tetQ*	Ap^R^ and Tc^R^, pUC19 derivative containing *tetQ* flanked by upstream and downstream regions of PGN_0725	This study
pKO1341-*erm*	Ap^R^ and Em^R^, pUC19 derivative containing the *erm* cassette flanked by upstream and downstream regions of PGN_1341	This study
pKO1888-*erm*	Ap^R^ and Em^R^, pUC19 derivative containing the *erm* cassette flanked by upstream and downstream regions of PGN_1888	This study
pKO1888-*tetQ*	Ap^R^ and Tc^R^, pUC19 derivative containing *tetQ* flanked by upstream and downstream regions of PGN_1888	This study
pKO1888-*cepA*	Ap^R^, pUC19 derivative containing *cepA* flanked by upstream and downstream regions of PGN_1888	This study
pGEX-6P-1	Ap^R^; GST fusion expression vector	GE Healthcare
pPR0725-GeX	Ap^R^; pGEX-6P-1 derivative containing PGN_0725 gene	This study
pPR1341-GeX	Ap^R^; pGEX-6P-1 derivative containing PGN_1341 gene	This study
pPR1888-GeX	Ap^R^; pGEX-6P-1 derivative containing PGN_1888 gene	This study
pPR1171-GeX	Ap^R^; pGEX-6P-1 derivative containing PGN_1171 gene	This study

### Construction of *P. gingivalis* Mutant Strains

*Porphyromonas gingivalis* deletion mutants lacking PGN_1171, PGN_0725, PGN_1341, and/or PGN_1888 gene were constructed by replacing each gene with *ermF* and *ermB* (*erm* cassette), as previously described (Yoshida et al., 2015). Approximately 0.7-kb-long upstream and downstream flanking fragments of each gene were PCR-amplified from genomic DNA of *P. gingivalis* ATCC 33277 using primers listed in Supplementary Table S1. The *erm* cassette was amplified from plasmid pVA2198 (Fletcher et al., 1997). The amplicons were joined using mixed PCR (Horton et al., 1989) or the In-fusion HD Cloning kit (Takara Bio, Otsu, Japan), and subcloned into pUC19 (GE Healthcare Japan, Hino, Japan). The resulting plasmids are listed in **Table 1**. Ligated fragments were introduced into *P. gingivalis* ATCC 33277 by electroporation. The constructed mutants were designated PGAGU101 (PGN_1171*::erm*), PGAGU104 (PGN_0725*::erm*), PGAGU108 (PGN_1888*::erm*), and PGAGU109 (PGN_1341*::erm*). To create double mutants, tetracycline resistance gene *tetQ*, amplified from plasmid pT-COW (Gardner et al., 1996), was used as an additional selectable marker. The *tetQ* gene and flanking fragments of the targeted genes were joined as described above, and used for electroporation of *P. gingivalis* single mutant strains to obtain PGAGU111 (PGN_0725*::erm* PGN_1888*::tetQ*), PGAGU114 (PGN_1341*::erm* PGN_1888*::tetQ*), or PGAGU115 (PGN_0725*::tetQ* PGN_1341*::erm*). A triple mutant strain lacking PGN_0725, PGN_1341, and PGN_1888 was also constructed, by replacing PGN_1888 in PGAGU115 with the ampicillin resistance gene *cepA*. The *cepA* gene was amplified from plasmid pCEPA, kindly provided by Dr. Naito (Nagasaki University, Nagasaki, Japan). The resultant mutant strain was designated PGAGU118 (PGN_0725*::tetQ* PGN_1341*::erm* PGN_1888*::cepA*). The integration of ligated PCR products at the expected chromosomal location(s) was confirmed by PCR amplification of the specific products across the upstream and downstream insertion boundaries using primers that were designed based on the flanking sequences extraneous to those used for gene targeting.

### Preparation of Crude Enzyme Extracts and Recombinant Proteins

Crude enzyme extracts were obtained from *P. gingivalis* ATCC 33277 and its derivatives grown anaerobically in modified GAM broth to log phase. Harvested cells were washed two times with phosphate-buffered saline (PBS), suspended in PBS containing 0.1 mM *N*-α-tosyl-L-lysine chloromethyl ketone, 0.2 mM phenylmethylsulfonyl fluoride, and 0.1 mM leupeptin (as protease inhibitors), and then lysed by ultrasonication on ice. After ultracentrifugation at 30,000 × *g* to remove insoluble material, crude enzyme concentrations were determined using the Pierce BCA Protein Assay Kit (Thermo Fisher Scientific, Rockford, IL, USA).

Recombinant PGN_0725, PGN_1341, PGN_1888, and PGN_1171 proteins were obtained using the expression vector pGEX-6P-1 (GE Healthcare Japan), as described previously (Yoshida et al., 2015). Coding sequences were PCR-amplified from the genomic DNA of *P. gingivalis* ATCC 33277 using primers listed in Supplementary Table S1. The amplicons were ligated into pGEX-6P-1 vector, and the resulting plasmids were verified by sequencing and are listed in **Table 1**. Protein concentrations were determined, as described previously (Pace et al., 1995), and protein purity was assessed by SDS-PAGE.

### Colorimetric Assay

CoA transferase activity in crude enzyme extracts or purified recombinant proteins was measured by determining the concentrations of acetyl-CoA, a reaction byproduct, with citrate synthase assay (Scherf and Buckel, 1991). Reaction mixtures (40 μl) consisted of 40 mM potassium phosphate buffer (pH 8.0), 200 mM sodium acetate, and 1 mM CoA derivatives (propionyl-CoA, butyryl-CoA, crotonyl-CoA, acetoacetyl-CoA, succinyl-CoA, or 3-hydroxybutyryl-CoA). After pre-warming, reactions were initiated by the addition of enzyme (25 μg/ml crude enzyme extracts, 125 ng/ml PGN_0725, 1.25 μg/ml PGN_1341, or 125 ng/ml PGN_1888), and incubated for 5 min. The reactions were terminated by the addition of 10 μl of 4.5% trichloroacetic acid. To neutralize the solution, 25 μl of 400 mM potassium phosphate buffer (pH 8.0) was added. After neutralization, assay mixtures (25 μl) containing 4 mM oxaloacetate, 4 mM 5,5′-dithiobis-2-nitrobenzoic acid (Sigma-Aldrich, St. Louis, MO, USA), and 36.8 μg/ml citrate synthase (Sigma-Aldrich) were added to the reaction mixtures for acetyl-CoA determinations. After incubation at 37°C for 30 min, the samples were examined spectrophotometrically at 412 nm (*A*_412_). Acetyl-CoA concentrations were calculated using a standard curve. In addition to the reaction conditions described above, varying concentrations of sodium acetate (20–250 mM) and either butyryl-CoA (0.5–5.0 mM) or propionyl-CoA (0.2–3.0 mM) were used to determine the kinetic parameters of each recombinant protein. These parameters were computed from Lineweaver–Burk transformation (*V*^-1^ vs. *S*^-1^) of the Michaelis–Menten equation. The *k*_cat_ values were calculated from *V*_max_ and molecular weights of the proteins. Data were obtained from three independent experiments.

Recombinant protein CoA transferase activity with 4-hydroxybutyrate and acetyl-CoA was determined using PGN_0724, a succinate semialdehyde reductase producing 4-hydroxybutyrate from succinate semialdehyde (Yoshida et al., 2015), since 4-hydroxybutyrate is not commercially available. Each reaction mixture (100 μl) contained 40 mM potassium phosphate buffer (pH 8.0), 0.5 mM succinate semialdehyde, 0.5 mM NADH, 0.5 mM acetyl-CoA, 1 mM MnCl_2_, and 1 μg/ml recombinant protein (PGN_0724, PGN_0725, PGN_1341, and/or PGN_1888). After incubation for 60 min at 37°C, the concentration of unconsumed NADH was determined spectrophotometrically at *A*_340_, using a standard curve. The concentration of unconsumed acetyl-CoA was determined using a citrate synthase assay, as described above.

### Gas Chromatography/Mass Spectrometry analysis

SCFAs in the reaction mixtures and culture supernatants were evaluated by gas chromatography/mass spectrometry (GC-MS) with a ZB-FFAP column (length, 30 m; diameter, 0.32 mm; film thickness, 0.25 μm; Phenomenex Inc., Torrance, CA, USA), as described previously (Yoshida et al., 2015). Briefly, the injection port temperature was set at 150°C. Helium was used as the carrier gas, with a linear velocity of 50 cm/s. The oven temperature was held at 80°C for 1 min, increased to 135°C at a rate of 10°C/min, further increased to a final temperature of 230°C at a rate of 30°C/min, and held for 6 min. For MS, the ionization source temperature was 200°C. The volume injected was 1 μl in splitless mode. To quantify the concentrations of individual SCFAs, experiments were performed in selected ion monitoring mode. Each enzyme reaction mixture (100 μl), containing 40 mM potassium phosphate buffer (pH 8.0), 500 ng/ml recombinant protein, 200 mM sodium acetate, and either 1 mM butyryl-CoA or 1 mM propionyl-CoA, was incubated at 37°C for 60 min. The bacterial supernatant samples were prepared by diluting the bacterial cultures with fresh modified GAM broth to optical density at 600 nm (OD_600_) = 0.375 ± 0.025 (1.5 × 10^5^ CFU/μl) to normalize cell concentration. Diluted bacterial cultures were immediately centrifuged to remove bacterial cells. Aliquots (100 μl) of enzyme reaction mixtures or supernatants were added to 500 μl of acetone. After incubation at -20°C for 2 h, the samples were centrifuged to remove proteins.

### Statistical Analysis

Differences between groups were analyzed by one-way analysis of variance, followed by the Student–Newman–Keuls multiple-comparisons test. Differences were considered significant when *P* < 0.01.

## Results

### Identification of PGN_0725, PGN_1341, and PGN_1888 Proteins as Butyryl-CoA:Acetate CoA Transferases

Although PGN_1171 has been previously annotated as a butyryl-CoA:acetate CoA transferase of *P. gingivalis* ATCC 33277 (Nelson et al., 2003; Hendrickson et al., 2009), the deduced PGN_1171 amino acid sequence shared no more than 20 and 27% identity with *R. hominis* RHOM_13820 protein and *Clostridium aminobutyricum* AbfT protein, respectively, both of which were experimentally shown to function as butyryl-CoA:acetate CoA transferases (Scherf and Buckel, 1991; Charrier et al., 2006). In addition, PGN_1171 (220 aa) was much shorter than RHOM_13820 (446 aa) and AbfT (438 aa). A database search revealed greater RHOM_13820 amino acid identity with PGN_0725 (38.5%), PGN_1341 (23.7%), and PGN_1888 (36.4%) proteins, all of which have been annotated as putative CoA transferases in *P. gingivalis* ATCC 33277 (Naito et al., 2008), than PGN_1171. Moreover, the lengths of PGN_0725 (431 aa), PGN_1341 (498 aa), and PGN_1888 (431 aa) proteins were similar to RHOM_13820 and AbfT.

To evaluate the function of PGN_1171, PGN_0725, PGN_1341, and PGN_1888 proteins, eight deletion strains, including double and triple mutants, were constructed using erythromycin, tetracycline, and ampicillin resistance genes (**Table 1**). Integration of the resistance genes at the expected chromosomal locations in the resulting mutant strains was verified by PCR (Supplementary Figure S1).

**FIGURE 2:**
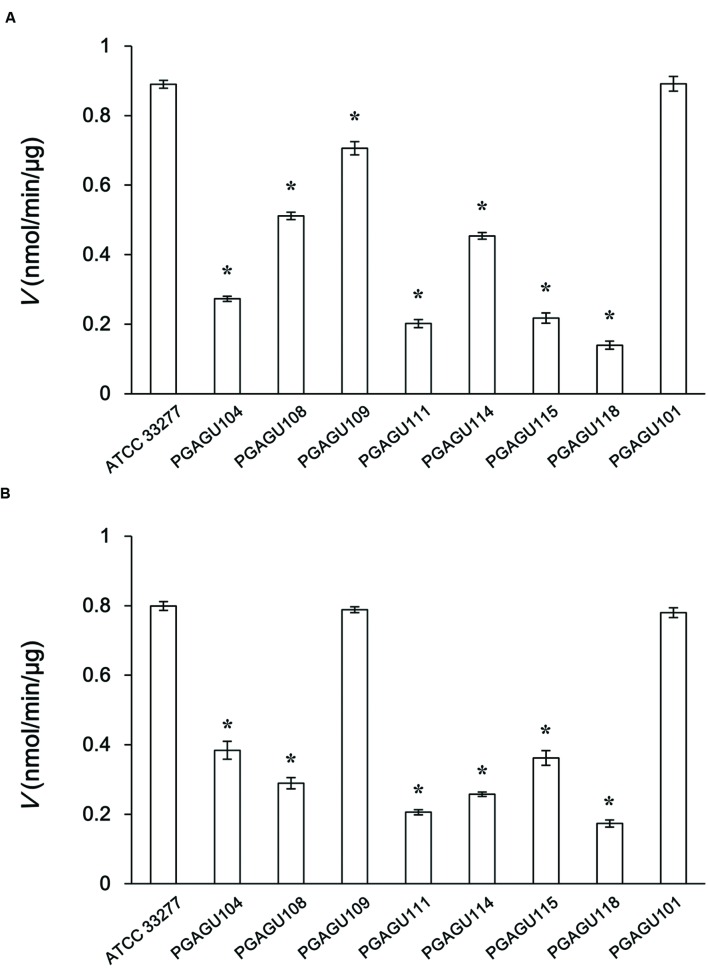
**Initial velocities of crude enzyme extracts of *P. gingivalis* ATCC 33277 and its mutant strains for butyryl-CoA **(A)** and propionyl-CoA **(B)****. The reaction mixtures, containing 25 μg/ml crude enzyme extracts, 200 mM sodium acetate, and 1 mM butyryl-CoA or propionyl-CoA, were incubated for 5 min. The concentration of CoA, a reaction byproduct, was measured. Data represent the mean ± standard deviation (*n* = 3). Asterisks indicate significant differences compared with the wild type (*P* < 0.01).

Butyrate and acetyl-CoA are produced from butyryl-CoA and acetate in the last step of butyrate production (**Figure 1**). Butyryl-CoA:acetate CoA transferase activity of crude enzyme extracts was determined by measuring the production of acetyl-CoA from butyryl-CoA and sodium acetate. The initial velocity of acetyl-CoA production by PGAGU101 (PGN_1171*::erm*) crude enzyme extracts was not significantly different from that of the wild type (**Figure 2A**), indicating that PGN_1171 has no detectable butyryl-CoA:acetate CoA transferase activity. By contrast, disruption of PGN_0725, PGN_1341, or PGN_1888 resulted in a significant decrease in the reaction velocity, suggesting that all the encoded proteins possess the butyryl-CoA:acetate CoA transferase activity.

Likewise, propionate and acetyl-CoA were produced from propionyl-CoA and acetate (**Figure 1**). The abilities of crude enzyme extracts of PGAGU101, PGAGU109 (PGN_1341*::erm*), and the wild type strain to form acetyl-CoA from propionyl-CoA and acetate were not significantly different (**Figure 2B**). The initial velocities of PGAGU104 (PGN_0725*::erm*) and PGAGU108 (PGN_1888*::erm*) crude enzyme extracts were significantly lower than that of the wild type. These findings demonstrated that PGN_0725 and PGN_1888, but not PGN_1171 and PGN_1341, possess the propionyl-CoA:acetate CoA transferase activity.

### Enzymatic Characterization of Recombinant PGN_0725, PGN_1341, and PGN_1888 Proteins

**FIGURE 3:**
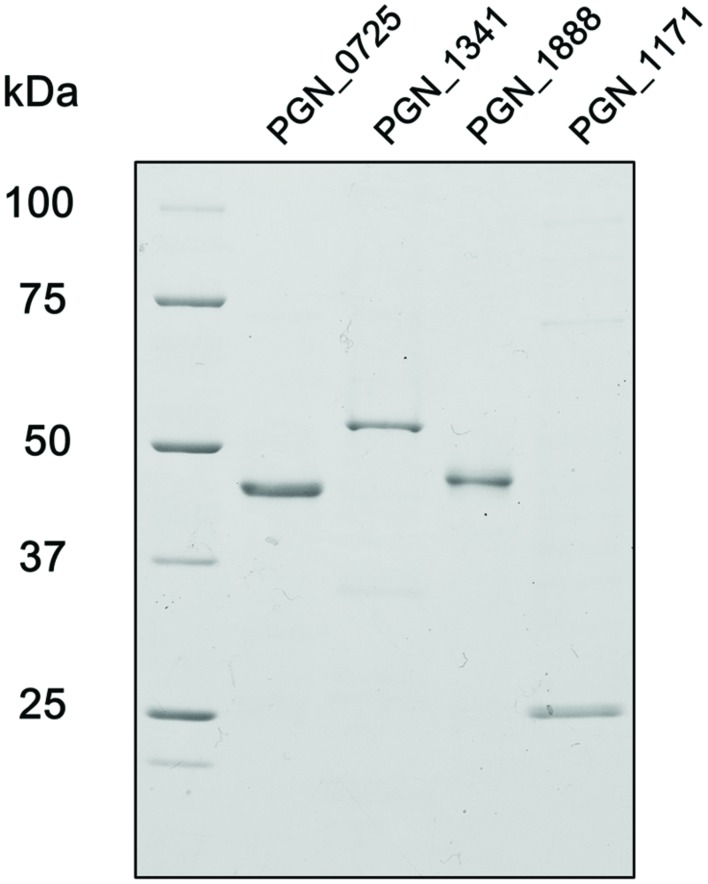
**SDS-PAGE analysis of recombinant *P. gingivalis* ATCC 33277 proteins**. After electrophoresis, samples (∼2 μg) were visualized by Coomassie Brilliant Blue staining. The positions of molecular mass markers (kDa) are shown.

To characterize PGN_0725, PGN_1341, PGN_1888, and PGN_1171 protein functions, each corresponding gene was expressed in *E. coli* to produce recombinant proteins. Molecular masses of the denatured PGN_0725, PGN_1341, PGN_1888, and PGN_1171 polypeptides agreed well with the respective predicted molecular masses (47.4, 54.7, 47.8, and 23.2 kDa, respectively; **Figure 3**).

**FIGURE 4:**
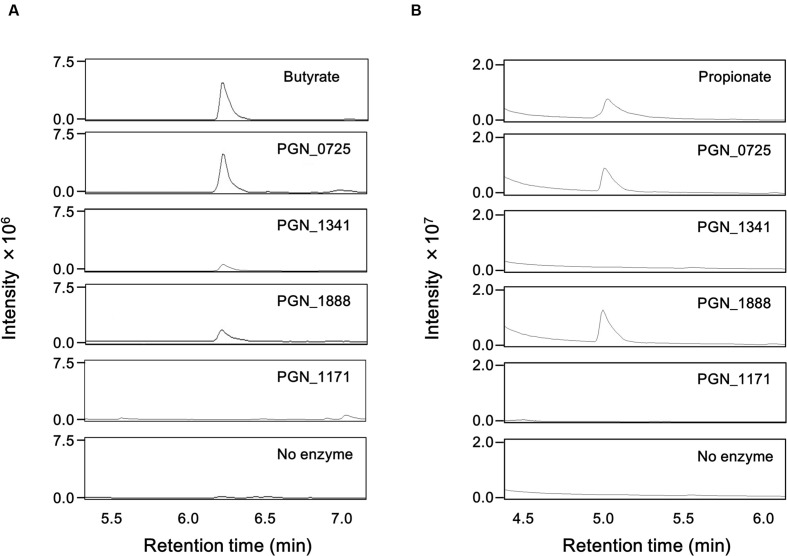
**GC-MS chromatograms of butyrate and propionate in reaction mixtures**. Total ion chromatogram of butyrate **(A)** and propionate **(B)** is shown. The mass spectra of each sample agree well with those of the corresponding standard. The reaction mixtures, containing 200 mM sodium acetate and either 1 mM butyryl-CoA or 1 mM propionyl-CoA, with recombinant PGN_0725, PGN_1341, PGN_1888, or PGN_1171 proteins, were incubated for 1 h. After the proteins were removed by acetone precipitation treatment, aqueous phase aliquot (1 μl) was analyzed.

GC-MS analysis demonstrated that incubation of butyryl-CoA and sodium acetate with any of the recombinant PGN_0725, PGN_1341, and PGN_1888 proteins led to the production of butyrate (**Figure 4**). Propionate was detected in reaction mixtures containing propionyl-CoA, sodium acetate, and either recombinant PGN_0725 or PGN_1888, but not PGN_1341. As expected, incubation of the recombinant PGN_1171 protein with butyryl-CoA or propionyl-CoA in the presence of sodium acetate resulted in no detectable production of butyrate or propionate, respectively (**Figure 4**).

**FIGURE 5:**
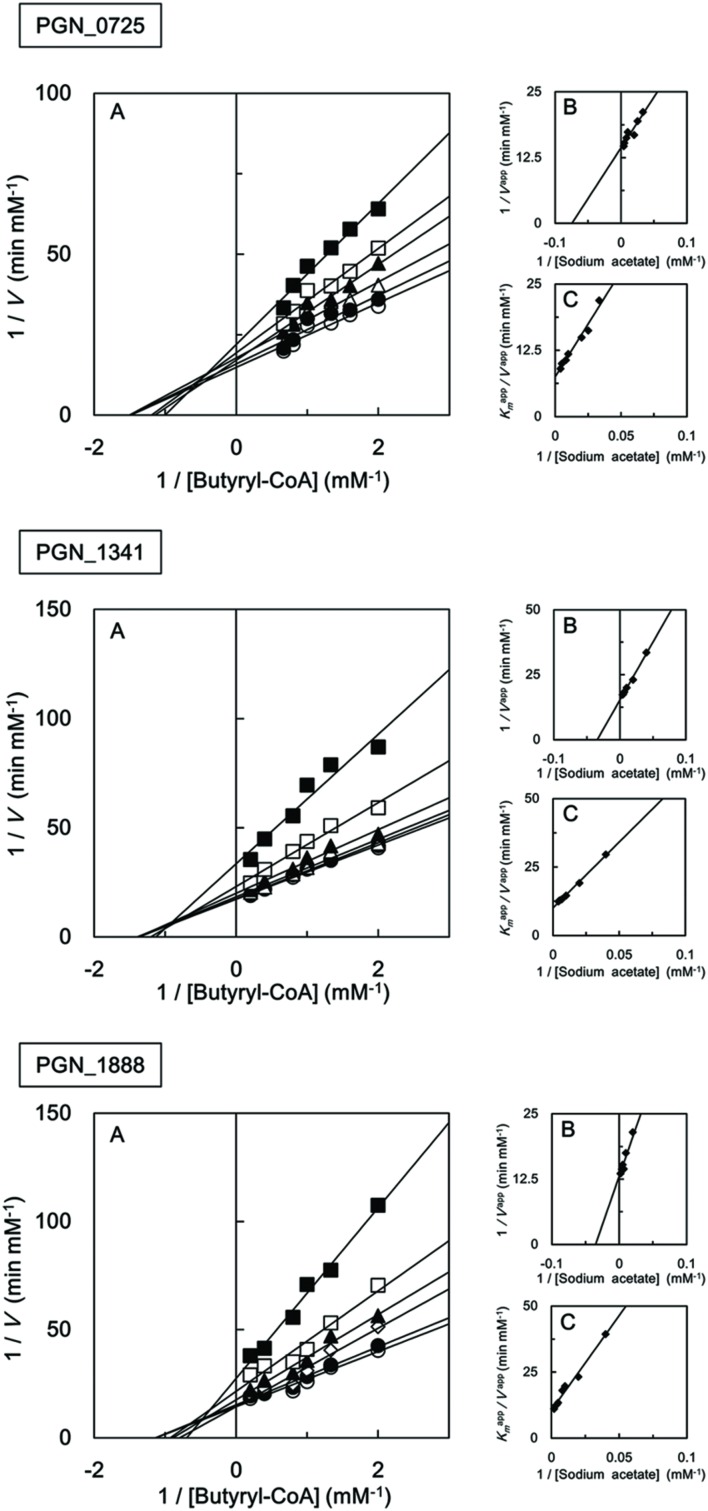
**Steady-state kinetic analysis of butyryl-CoA:acetate CoA transferase activities of recombinant PGN_0725, PGN_1341, and PGN_1888 proteins**. **(A)** Double reciprocal plots of the initial velocities of acetyl-CoA and butyrate formation from butyryl-CoA and sodium acetate catalyzed by purified recombinant enzymes. Different butyryl-CoA concentrations (0.5–5 mM) were assayed at fixed sodium acetate concentrations (25 mM, filled squares; 50 mM, open squares; 100 mM, filled triangles; 125 mM, open triangles; 200 mM, filled circles; or 250 mM, open circles). **(B)** Secondary plots of *y* intercepts (velocity reciprocals) vs. sodium acetate concentrations. **(C)** Secondary plots of the reciprocal slopes from panel **(A)** vs. sodium acetate concentration. Data represent the mean ± standard deviation (*n* = 3).

**FIGURE 6:**
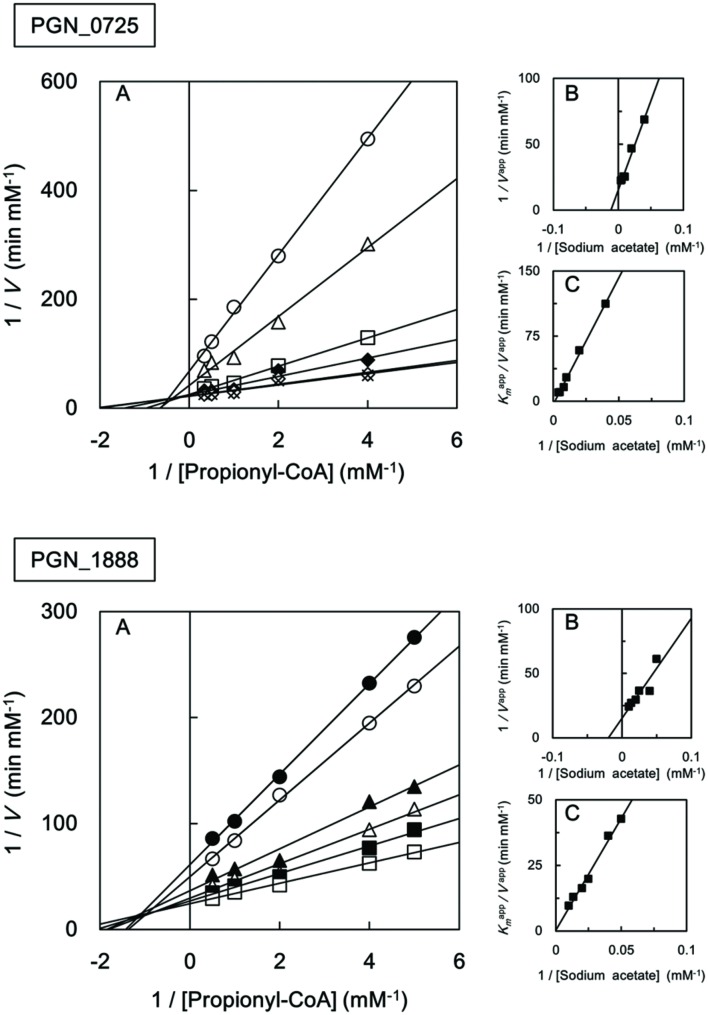
**Steady-state kinetic analysis of propionyl-CoA:acetate CoA transferase activities of recombinant PGN_0725 and PGN_1888 proteins**. **(A)** Double reciprocal plots of the initial velocities of acetyl-CoA and propionate formation from propionyl-CoA and sodium acetate catalyzed by purified recombinant enzymes. Different concentrations of propionyl-CoA (0.2–3 mM) were assayed at fixed sodium acetate concentrations (20 mM, filled circles; 25 mM, open circles; 40 mM, filled triangles; 50 mM, open triangles; 75 mM, filled squares; 100 mM, open squares; 125 mM, filled diamonds; 200 mM, open diamonds; and 250 mM, crosses). **(B)** Secondary plots of *y* intercepts (velocity reciprocals) vs. sodium acetate concentrations. **(C)** Secondary plots of the reciprocal slopes from panel **(A)** vs. sodium acetate concentrations. Data represent the mean ± standard deviation (*n* = 3).

We also investigated the kinetic parameters of recombinant PGN_0725, PGN_1341, and PGN_1888 using a colorimetric assay. Initial velocities were determined at either fixed sodium acetate concentrations at different butyryl-CoA concentrations, or fixed butyryl-CoA concentrations at different sodium acetate concentrations. Lineweaver–Burk (double reciprocal) plots were then constructed (**Figure 5**). Line series intersecting to the left of *y*-axes (*V*^-1^) suggested that PGN_0725, PGN_1341, and PGN_1888 proteins act *via* a ternary-complex kinetic mechanism. *K*_m_ and *V*_max_ values were estimated from secondary plots (**Figures 5B,C**; **Table 2**). In addition, *k*_cat_ values were calculated from enzyme concentrations in the reaction mixtures. Kinetic properties of the recombinant PGN_0725 and PGN_1888 proteins were also determined by analyzing acetyl-CoA production from sodium acetate and propionyl-CoA (**Table 3**). These reactions also follow the ternary-complex mechanism (**Figure 6**).

**Table 2:** Kinetic properties of recombinant PGN_0725, PGN_1341, and PGN_1888 proteins for butyryl-CoA and sodium acetate.

	PGN_0725	PGN_1341	PGN_1888
***K*_m_**			
Butyryl-CoA (μM)	520 ± 10	610 ± 40	920 ± 70
Sodium acetate (mM)	16 ± 1.8	26 ± 2.8	33 ± 5.3
*V*_max_ (μM min^-1^)	71 ± 0.5	65 ± 0.8	80 ± 3.3
*k*_cat_ (s^-1^)	560 ± 4.1	24 ± 0.3	254 ± 10

**Table 3:** Kinetic properties of recombinant PGN_0725 and PGN_1888 proteins for propionyl-CoA and sodium acetate.

	PGN_0725	PGN_1888
***K*_m_**		
Propionyl-CoA (μM)	108 ± 47	23 ± 4.4
Sodium acetate (mM)	92 ± 7.9	89 ± 23
*V*_max_ (μM min^-1^)	67.5 ± 4.0	86 ± 30
*k*_cat_ (s^-1^)	533 ± 31	1374 ± 477

### Substrate Specificity of Recombinant PGN_0725, PGN_1341, and PGN_1888 Proteins

To investigate substrate specificity of the recombinant proteins, four CoA derivatives (crotonyl-CoA, acetoacetyl-CoA, succinyl-CoA, and 3-hydroxybutyryl-CoA) were evaluated as substrates, in addition to butyryl-CoA and propionyl-CoA (Supplementary Figure S2). When the CoA derivatives tested were incubated with PGN_0725, PGN_1341, or PGN_1888 in the presence of sodium acetate, no significant production of acetyl-CoA was detected, suggesting that crotonyl-CoA, acetoacetyl-CoA, succinyl-CoA, and 3-hydroxybutyryl-CoA were not used as a substrate by these enzymes.

Next, sodium butyrate and sodium propionate (instead of sodium acetate) were tested as enzyme substrates in the presence of propionyl-CoA and butyryl-CoA, respectively. GC-MS analysis revealed that the incubation of a sodium butyrate/propionyl-CoA pair or sodium propionate/butyryl-CoA pair with PGN_0725, PGN_1341, or PGN_1888 resulted in no detectable production of propionate or butyrate (data not shown). These findings suggested that the three CoA transferases utilize no SCFA substrates other than acetate.

Since PGN_0725 was annotated as a 4-hydroxybutyrate CoA transferase (Nelson et al., 2003; Naito et al., 2008; Hendrickson et al., 2009), an enzyme catalyzing the formation of 4-hydroxybutyryl-CoA from 4-hydroxybutyrate and acetyl-CoA (**Figure 1**), we examined the 4-hydroxybutyrate CoA transferase activity of recombinant PGN_0725 (Supplementary Figure S3). In this assay, the recombinant PGN_0724 that produces 4-hydroxybutyrate from succinate semialdehyde was used (Yoshida et al., 2015), because the 4-hydroxybutyrate is not commercially available. When recombinant PGN_0724 was incubated with NADH and succinate semialdehyde, NADH was consumed, indicating 4-hydroxybutyrate production. However, incubation of a mix of recombinant PGN_0724 and PGN_0725 proteins with the reaction mixture resulted in no significant acetyl-CoA consumption, suggesting that PGN_0725 does not produce 4-hydroxybutyryl-CoA from 4-hydroxybutyrate and acetyl-CoA. Incubation of either recombinant PGN_1341 or PGN_1888 (instead of PGN_0725) in the reaction mixture did not result in acetyl-CoA consumption (data not shown). These findings suggested that PGN_0725, PGN_1341, and PGN_1888 did not function as 4-hydroxybutyrate CoA transferases.

### Production of SCFAs by *P. gingivalis* ATCC 33277 and Mutant Strains

**FIGURE 7:**
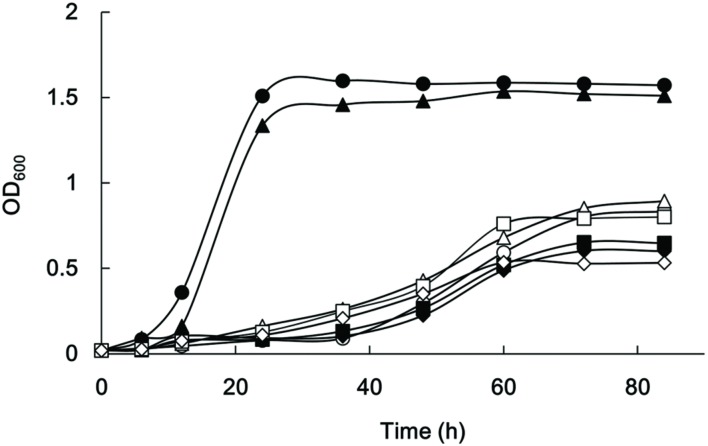
**Growth of *P. gingivalis* strains**. *P. gingivalis* ATCC 33277 (filled circles), PAGU104 (open circles), PAGU108 (filled triangles), PAGU109 (open triangles), PAGU111 (filled squares), PAGU114 (open squares), PAGU115 (filled diamonds), and PAGU118 (open diamonds) were assessed after 6, 12, 24, 36, 48, 60, 72, and 84 h. Representative data for at least two independent experiments are shown.

PGAGU108 grew as fast as the wild type, as determined by OD_600_ measurements of the cell cultures (**Figure 7**). By contrast, the growth rates of other mutant strains were obviously slower than that of the wild type, as were culture turbidities in the stationary phase. This suggested that the intermediates and end products of the butyrate, propionate, and related molecule biosynthetic pathways might be important for the metabolism of this microorganism.

**FIGURE 8:**
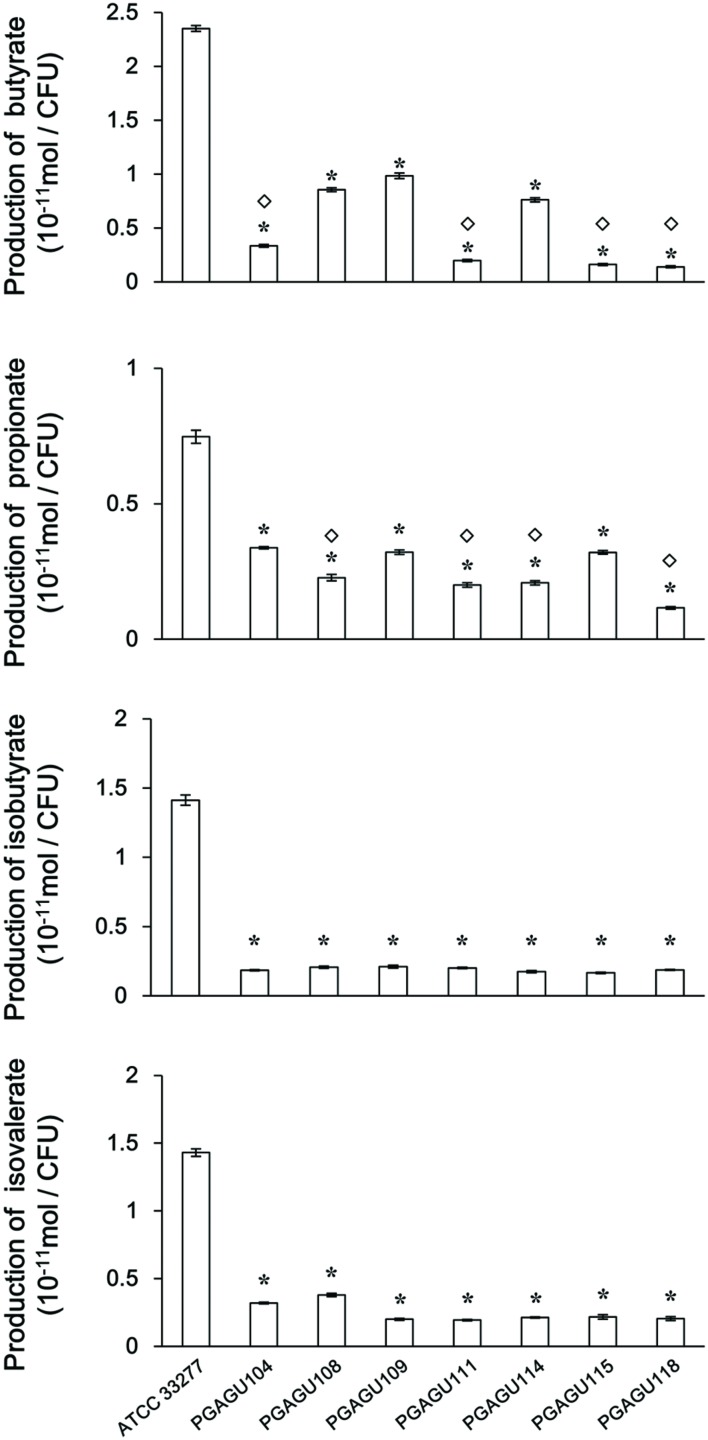
**SCFA concentrations in culture supernatants of *P. gingivalis* ATCC 33277 and mutant strains**. The supernatants were obtained by centrifugation of bacterial cultures (OD_600_ 0.375 ± 0.025) and diluted 1:6. Data represent the mean ± standard deviation (*n* ≥ 3). Asterisks indicate significant differences compared with the wild type (*P* < 0.01). Diamonds indicate significant differences compared with each no diamond strain (*P* < 0.01).

To evaluate the contribution of PGN_0725, PGN_1341, and PGN_1888 to SCFA production, SCFA concentrations in stationary phase bacterial cultures were quantified using GC-MS. The OD_600_ of each culture was adjusted to 0.375 ± 0.025 to normalize cell concentrations. This conversion allowed us to compare the production of SCFAs per cell number in the wild type and mutant strains (**Figure 8**). Butyrate concentrations in culture media of all the mutant strains were significantly lower than that of the wild type (*P* < 0.01). Butyrate concentrations in the culture supernatants of PGAGU104, PGAGU111 (PGN_0725*::erm* PGN_1888*::tetQ*), PGAGU115 (PGN_0725*::tetQ* PGN_1341*::erm*), and PGAGU118 (PGN_0725*::tetQ* PGN_1341*::erm* PGN_1888*::cepA*), all of which lacked the PGN_0725 gene, were significantly lower, not only than that of the wild type, but also than those of other mutants. Likewise, propionate concentrations in culture supernatants of PGAGU108, PGAGU111, PGAGU114 (PGN_1341*::erm* PGN_1888*::tetQ*), and PGAGU118, all of which lacked the PGN_1888 gene, were significantly lower than those of other strains. Deletion of PGN_0725, PGN_1341, or PGN_1888 resulted in a drastic decrease in isobutyrate and isovalerate concentrations in culture supernatants (**Figure 8**). Interestingly, the triple mutant, PGAGU118, still released butyrate, propionate, isobutyrate, and isovalerate into the culture supernatant (**Figure 8**).

## Discussion

In the current study, we identified and characterized three CoA transferases, PGN_0725, PGN_1341, and PGN_1888, by a homology search with CoA transferase in *R. hominis* (Charrier et al., 2006; Travis et al., 2015). All the proteins catalyze the production of butyrate and acetyl-CoA from butyryl-CoA and acetate in the butyrate synthesis pathway of *P. gingivalis*. In addition, PGN_0725 and PGN_1888 proteins also produced propionate and acetyl-CoA from propionyl-CoA and acetate. Despite previous annotation of PGN_1171 as a protein responsible for this reaction to produce butyrate (Nelson et al., 2003; Hendrickson et al., 2009), the protein had no detectable butyryl-CoA:acetate CoA transferase or propionyl-CoA:acetate CoA transferase activity (**Figure 2**). The deduced amino acid sequence of PGN_1171 shared 46 and 24% identity with PcaJ from *Pseudomonas putida* (Parales and Harwood, 1993) and *Sinorhizobium meliloti* (MacLean et al., 2006), respectively. The PcaJ protein comprises one of the two subunits of succinyl-CoA/β-ketoadipate CoA transferase that catalyze the production of β-ketoadipyl-CoA from β-ketoadipate and succinyl-CoA. PGN_1171 might be a subunit of a heterodimer functioning as a CoA transferase for CoA derivatives other than butyryl-CoA and propionyl-CoA. However, the function of PGN_1171 protein remains unknown.

Several mutant strains were constructed to examine the role of PGN_0725, PGN_1341, and PGN_1888 (**Table 1**; Supplementary Figure S1). Butyrate concentration in culture supernatants of PGAGU104, PGAGU111, PGAGU115, and PGAGU118 decreased by 85.7, 91.6, 93.1, and 94.1%, respectively, compared with that of the wild type strain (**Figure 8**). Notably, butyrate concentrations in culture supernatants of these mutants, all of which lacked PGN_0725 (**Table 1**), were significantly lower, not only than that of the wild type, but also other mutants. Similarly, butyryl-CoA:acetate CoA transferase activities in crude enzyme extracts of PGN_0725-lacking strains were lower than those of other strains (**Figure 2**). Furthermore, of the recombinant proteins tested, PGN_0725 displayed the highest butyryl-CoA:acetate CoA transferase activity (**Table 2**). Taken together, the PGN_0725 protein contributes the most to butyrate production in *P. gingivalis*. Likewise, the findings summarized in **Figures 2** and **8**, and **Table 3**, demonstrate that PGN_1888 is a CoA transferase contributing the most to the production of propionate. Deletion of PGN_0725, PGN_1341, or PGN_1888 resulted in low concentrations of isobutyrate and isovalerate in culture supernatants (**Figure 8**). These observations suggested that the isobutyrate and isovalerate synthetic pathways might share their intermediates with the butyrate synthetic pathway in *P. gingivalis*. Synthetic pathways of those molecules remain to be elucidated. It is also of interest that the CoA transferase activity in crude enzyme extracts of the triple mutant strain (PGAGU118) was ∼20% that of the wild type, suggesting that *P. gingivalis* harbors additional CoA transferase(s) other than PGN_0725, PGN_1341, and PGN_1888. CoA transferase(s) with relatively broad substrate specificity may be partially involved in the formation of butyrate and propionate from butyryl-CoA and propionyl-CoA, respectively. Further studies are necessary to precisely understand the production of butyrate and propionate in bacteria.

PGN_0725 was annotated as a 4-hydroxybutyrate CoA transferase (Nelson et al., 2003; Naito et al., 2008; Hendrickson et al., 2009). Indeed, the amino acid identity shared by PGN_0725 and *C. aminobutyricum* AbfT proteins, the latter of which was experimentally verified as a 4-hydroxybutyrate CoA transferase (Scherf and Buckel, 1991; Gerhardt et al., 2000), was higher than that shared by PGN_0725 and *R. hominis* RHOM_13820 protein, which presumably does not function as a 4-hydroxybutyrate CoA transferase (Charrier et al., 2006). However, no 4-hydroxybutyrate CoA transferase activity was detected in PGN_0725 recombinant protein (Supplementary Figure S3), similarly to the recombinant PGN_1341 and PGN_1888 proteins. Our findings also suggest that an additional CoA transferase(s) possibly exists in *P. gingivalis*. This study demonstrated that PGN_1341 functions as a CoA transferase producing butyrate from butyryl-CoA (**Figure 4**). However, considering that the butyryl-CoA:acetate CoA transferase activity of PGN_1341 is much lower than that of PGN_0725, the primary role of PGN_1341 in *P. gingivalis* remains unknown. Although PGN_1341 is not responsible for propionate production from propionyl-CoA in this bacterium, replacement of its gene with the *erm* cassette resulted in a reduction of propionate levels in culture supernatants (**Figure 8**). These findings suggest that PGN_1341 might primarily function as a CoA transferase involved in the butyrate and propionate biosynthetic pathways.

The *k*_cat_ values of *R. hominis* RHOM_13820 (Charrier et al., 2006) and *C. aminobutyricum* AbfT (Gerhardt et al., 2000) for butyryl-CoA (91.5 and 119 s^-1^, respectively) were higher than that of PGN_1341, but lower than those of PGN_0725 and PGN_1888. By contrast, *k*_cat_ values of RHOM_13820 and AbfT for propionyl-CoA (41.7 and 120 s^-1^, respectively) were also much lower than those of PGN_0725 and PGN_1888 proteins. PGN_0725, PGN_1341, and PGN_1888 proteins catalyzed transferase reactions via a ternary-complex kinetic mechanism, whereas RHOM_13820 protein was reported to catalyze a transferase reaction via a ping-pong bi-bi mechanism (Charrier et al., 2006). Thus, PGN_0725, PGN_1341, and PGN_1888 were different from AbfT in terms of substrate specificity and different from RHOM_13820 in terms of kinetic mechanism. Clarification of the precise enzymatic mechanisms underlying enzyme binding of the butyryl-CoA or propionyl-CoA substrates might require crystallographic analyses.

The concentrations of propionate and butyrate in the gingival crevices are associated significantly with clinical measures of the periodontal diseases’ severity and inflammation (Niederman et al., 1997). Nevertheless, a database search analysis showed that the orthologs of PGN_0725, PGN_1341, and PGN_1888 are only in limited species of anaerobic periodontopathogens, including *Tannerella forsythia* and *Fusobacterium nucleatum*, but not in *Treponema denticola* and *Prevotella intermedia*, both of which are representative periodontopathogens (Socransky and Haffajee, 2002). The relationship between the clinical severity and the proportion of the SCFA-producing bacteria in the gingival crevices has to be investigated to understand the effect of SCFAs in the gingival crevices on the initiation and progression of periodontitis.

## Conclusion

This study addressed the function of three genes, and the proteins that they encode, as CoA transferases associated with SCFAs production in *P. gingivalis*. PGN_0725, PGN_1341, and PGN_1888 were involved in the last step of butyrate production, where butyrate and acetyl-CoA were produced from acetate and butyryl-CoA. PGN_0725 and PGN_1888 also catalyze the transfer of CoA moieties of propionyl-CoA to acetate, to produce propionate. Culture supernatant of the triple deletion mutant still contained a small amount of butyrate and propionate, suggesting the existence of additional CoA transferase(s). Further studies of molecules associated with the butyrate production pathway are currently underway in our laboratory.

## Author Contributions

Conception and design of the experiments: YY. Acquisition of the data: MS, YY. Analysis of the data: MS, YY, KN, YH. Interpretation of data: MS, YY, JT, FY.

## Conflict of Interest Statement

The authors declare that the research was conducted in the absence of any commercial or financial relationships that could be construed as a potential conflict of interest.

## ABBREVIATIONS

OD600: optical density at 600 428 nm
PBS: phosphate buffered saline
SCFAs: short chain fatty acids
