# Supplementary material for: Three CoA Transferases Involved in the Production of Short Chain Fatty Acids in Porphyromonas gingivalis
Source: Front Microbiol. 2016 Jul 19;7:1146. doi: 10.3389/fmicb.2016.01146 (PMC4949257; doi:10.3389/fmicb.2016.01146)
Supplement: Supplementary file 1 [file Table_1.DOCX]

Table S1. Oligonucleotide primes used in this study

| Purpose | Designation | Primer | Sequence (5' to 3') ^*^ |
| --- | --- | --- | --- |
| Construction of mutant strain |  |  |  |
|  | the *erm* cassette | 012613-ermFAM-F1 | CCGATAGCTTCCGCTATTG |
|  |  | 033012-ermFAM-R1 | CGACTCATAGAATTATTTCCTCC |
|  | *tetQ* | 122414-tetQ-F | AAACGCTATACCGAGAGAGAAAC |
|  |  | 122414-tetQ-R | CCAACCGTATTGCCTTATAGAAAT |
|  | *cepA* | 120115-cepA-F | AAAAGAGTTAAGGAAAGTGAAGC |
|  |  | 120115-cepA-R | TTTCAAGTCACCGATAGTG |
|  | Upstream region of PGN_1171 | 021513-PGN1171-F2 | ACACGATGAATGACGGTAAG |
|  |  | 021513-PGN1171-R1-erm | CAATAGCGGAAGCTATCGGGACTACATCACCATCTTTCAATTC |
|  | Downstream region of PGN_1171 | 021513-PGN1171-F3-erm | GGAGGAAATAATTCTATGAGTCGCTGATTATCACAGAAATGTGTGTG |
|  |  | 021513-PGN1171-R3 | GGCAAATACCACGTATACGT |
|  | Upstream region of PGN_0725 | 072313-0725KO-F-infusion | AAAACGACGGCCAGTGAATTCCTTATGAAATAGCTCGCTTG |
|  |  | 061213-0725KO-R1-erm | CAATAGCGGAAGCTATCGGATGTGACAAAGCTACCCGTT |
|  |  | 061015-0725KO-R1-tet | GTTTCTCTCTCGGTATAGCGTTTATGTGACAAAGCTACCCGTT |
|  | Downstream region of PGN_0725 | 061213-0725KO-F3-erm | GGAGGAAATAATTCTATGAGTCGTCTCGAATTGTACCTATAATTGCA |
|  |  | 061015-0725KO-F3-tet | ATTTCTATAAGGCAATACGGTTGGTCTCGAATTGTACCTATAATTGCA |
|  |  | 072313-0725KO-R-infusion | GACCATGATTACGCCAAGCTTGAATACTTCCAGGATAATGTCC |
|  | Upstream region of PGN_1341 | 102814-1341KO-F-infusion | AAAACGACGGCCAGTGAATTTACCCCGATAACGAGACGG |
|  |  | 102814-1341KO-R1-erm | CAATAGCGGAAGCTATCGGGCCGCAGGTACTACCTTAG |
|  | Downstream region of PGN_1341 | 102814-1341KO-F3-erm | GGAGGAAATAATTCTATGAGTCGTATCTGGAGCTTGGAGTCA |
|  | | 102814-1341KO-R-infusion | GACCATGATTACGCCAAGCTGACATGGATGCTCCTGCC |
|  | Upstream region of PGN_1888 | 102814-1888KO-F-infusion | AAAACGACGGCCAGTGAATTCTCAGCGTCTGGAACTCA |
|  |  | 102814-1888KO-R1-erm | CAATAGCGGAAGCTATCGGGACAGGCGCAGCAGCAGCA |
|  |  | 122414-1888KO-R1-tetQ | GTTTCTCTCTCGGTATAGCGTTTGACAGGCGCAGCAGCAGCA |
|  |  | 120115-1888KO-R1-cepA | GCTTCACTTTCCTTAACTCTTTTGACAGGCGCAGCAGCAGCA |
|  | Downstream region of PGN_1888 | 102814-1888KO-F3-erm | GGAGGAAATAATTCTATGAGTCGGAATATGGCGTAGCGCGTCT |
|  |  | 122414-1888KO-F3-tetQ | ATTTCTATAAGGCAATACGGTTGGGAATATGGCGTAGCGCGTCT |
|  |  | 120115-1888KO-F3-cepA | CACTATCGGTGACTTGAAAGAATATGGCGTAGCGCGTCT |
|  |  | 102814-1888KO-R-infusion | GACCATGATTACGCCAAGCTTCGACGTAATGTTACAGT |
|  |  |  |  |
| Construction of recombinant protein |  |  |  |
|  | PGN_0725 | 080513-pGN0725-F-Eco | **AAGAATTC**AAAGACGTATTAGCGGAATATGC |
|  |  | 072913-pGN0725-R-Sal | **TTGTCGAC**TTATCCGAAACGTTTGCG |
|  | PGN_1341 | 102814-pGN1341F-Eco | **AAGAATTC**GCTCTAAGATTTATCACTGCAGA |
|  |  | 102814-pGN1341R-Sal | **AAGTCGAC**TTACTTCATGTAGTCTTCCCAGC |
|  | PGN_1888 | 102814-pGN1888F-Eco | **AAGAATTC**CAATGGCAAGAACTTTACCGTCA |
|  |  | 102814-pGN1888R-Sal | **AAGTCGAC**TTATTCGAAGCGTCGGCGGAT |
|  | PGN_1171 | PGN1171-F1-Bam | **AAGGATCC**GAAAAAGATCAAATCAGAGAAGTG |
|  |  | PGN1171-R1-Sal | **AAGTCGAC**TTATTGTTGCATCGGGATG |

^*^ Underlined and doubly-underlined nucleotides indicate overlapping regions of the antibiotics resistant genes (the *erm* cassette, *tetQ*, and *cepA*)*,* and of the multi-cloning sites in pUC19, respectively. Bold nucleotides indicate the positions of restriction endonuclease sites incorporated to facilitate cloning into pGEX-6P-1.
