# Supplementary material for: Three CoA Transferases Involved in the Production of Short Chain Fatty Acids in Porphyromonas gingivalis
Source: Front Microbiol. 2016 Jul 19;7:1146. doi: 10.3389/fmicb.2016.01146 (PMC4949257; doi:10.3389/fmicb.2016.01146)
Supplement: Supplementary file 2 [file Presentation_1.PDF]

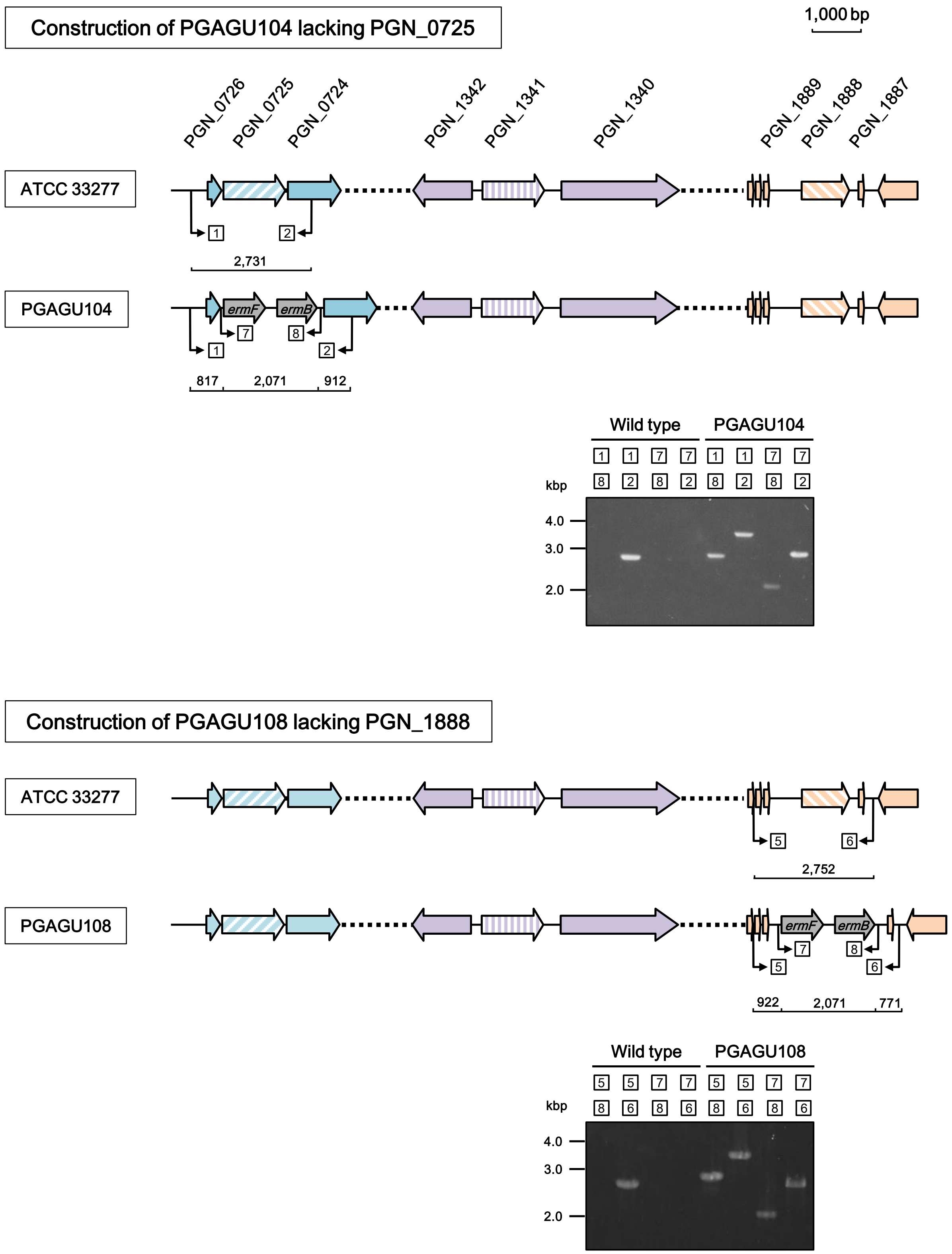

Figure S1. Mutant strain construction. For the construction of mutant strains, PGN_0725, PGN_1341, PGN_1888, and/or PGN_1171 genes were replaced by *erm* cassette, *tetQ*, and/or *cepA*. Each boxed number indicates a PCR primer used for construct verification. The size of each fragment (bp) is shown. Inset: Mutant verification by agarose gel electrophoresis. Each DNA fragment was PCR-amplified using the indicated primers. DNA size standards are shown.

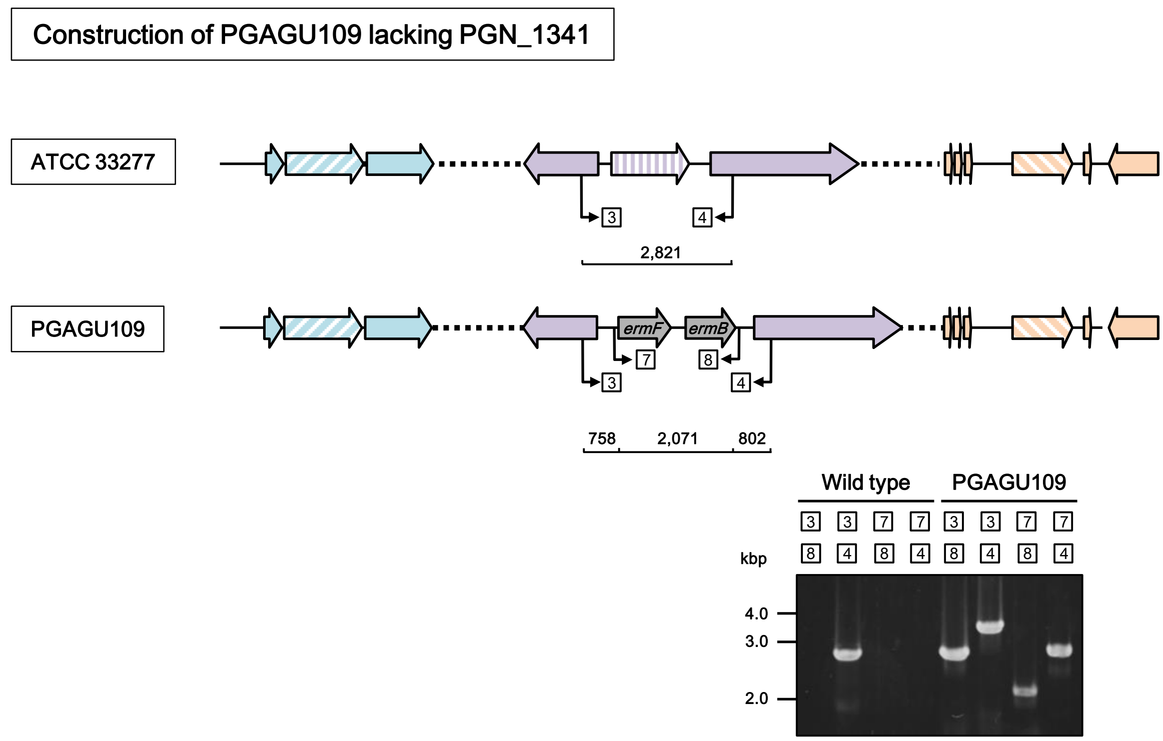

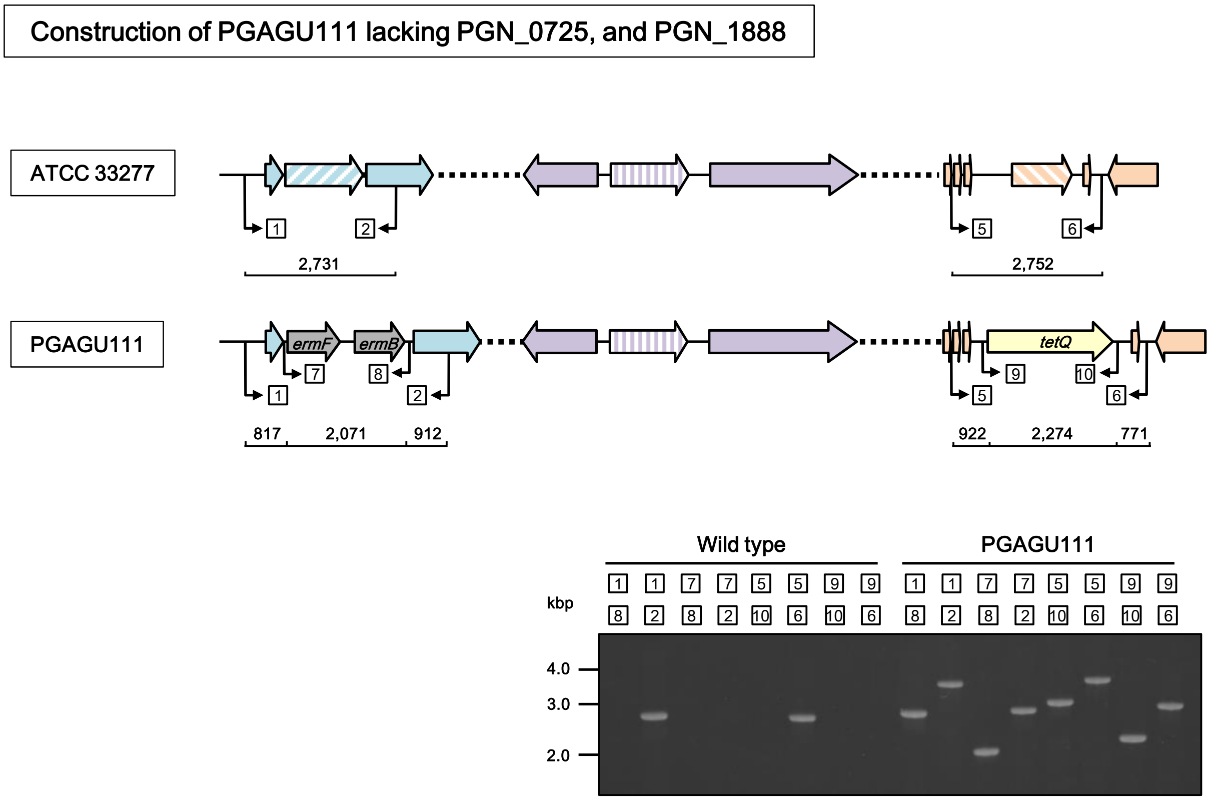

Fig. S1. Continued-1.

Construction of PGAGU114 lacking PGN_1341, and PGN_1888

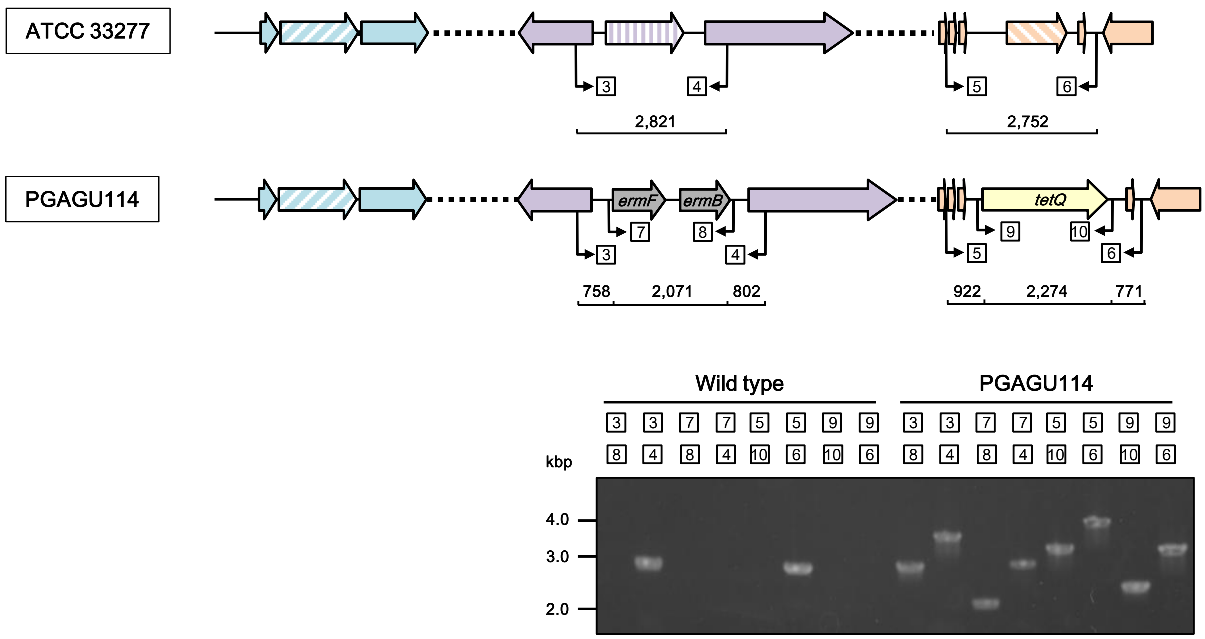

Construction of PGAGU115 lacking PGN_0725, and PGN_1341

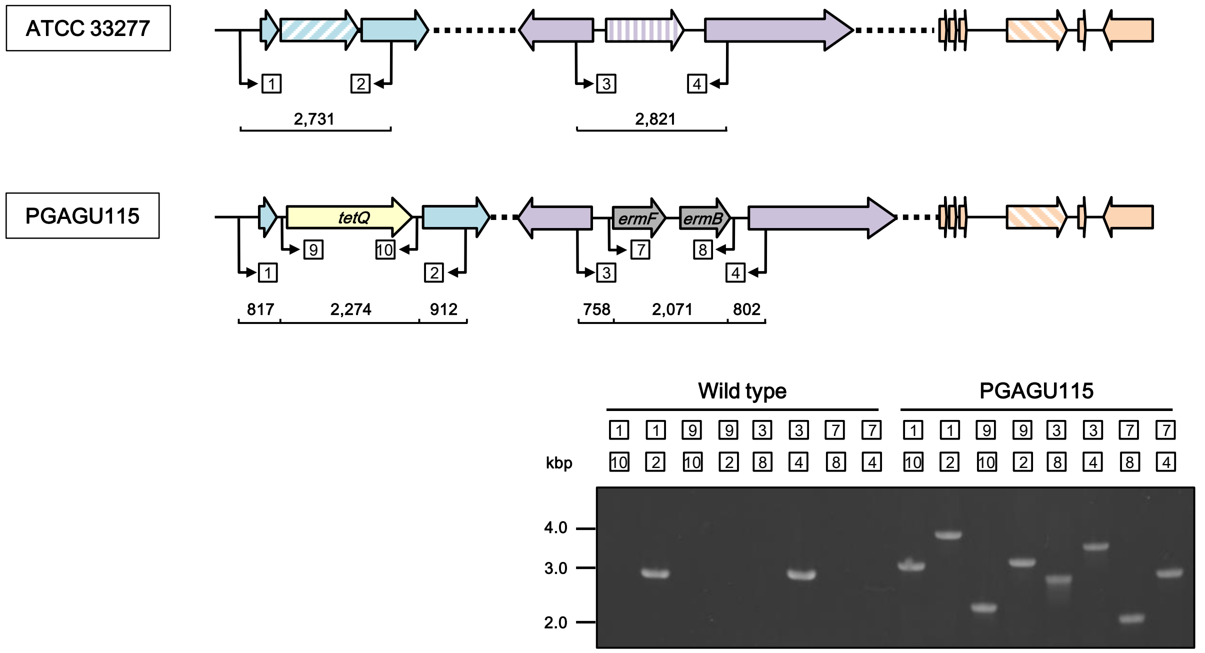

Fig. S1. Continued-2.

Construction of PGAGU118 lacking PGN_0725, PGN_1341, and PGN_1888

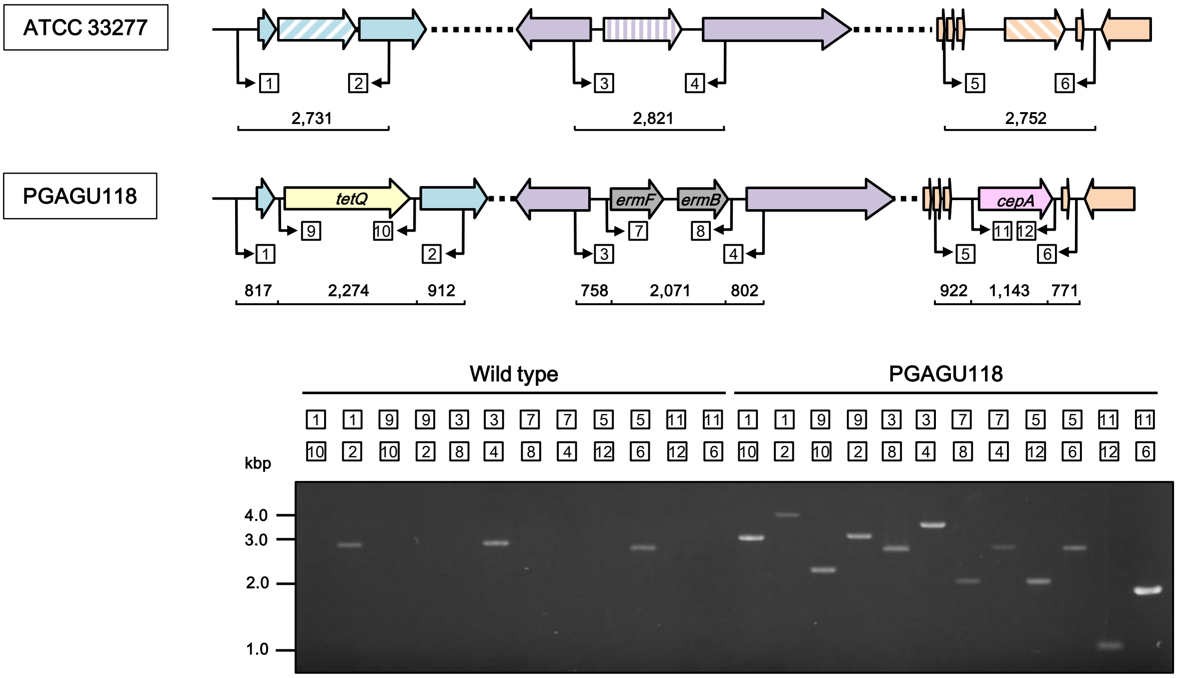

Construction of PGAGU101 lacking PGN_1171

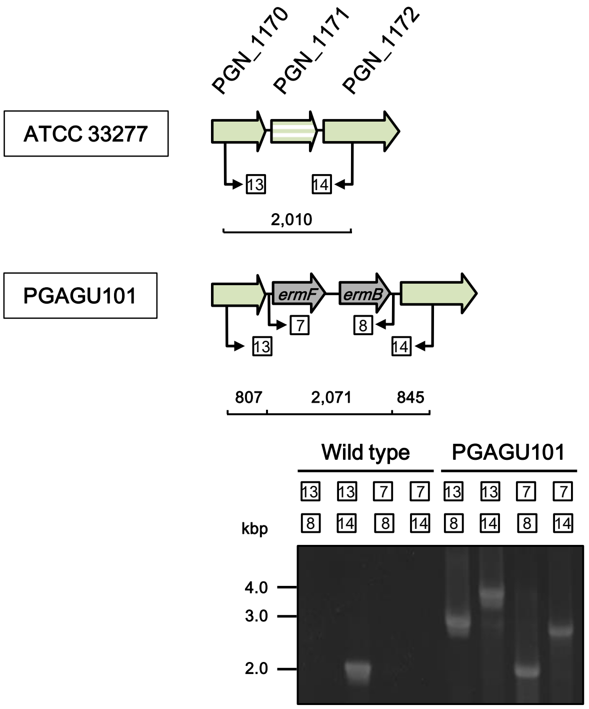

Fig. S1. Continued-3.

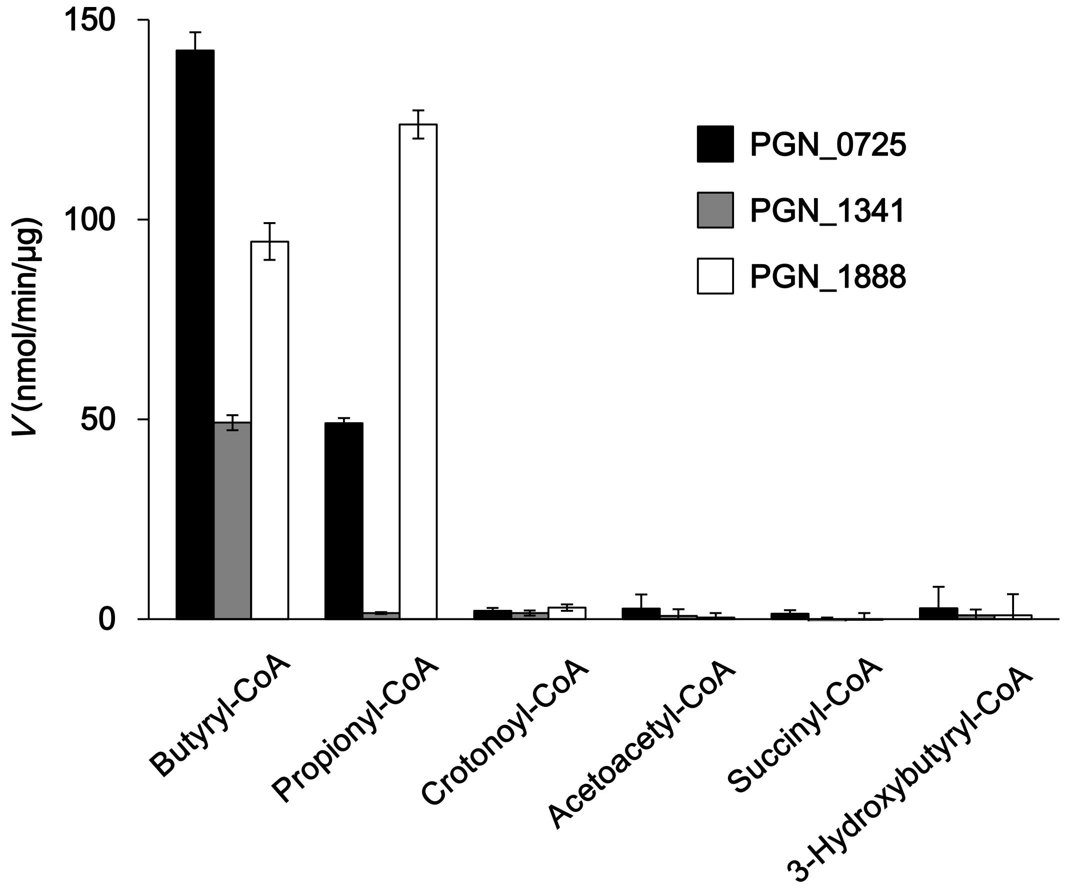

Fig. S2. Initial velocity of the recombinant enzymes for several CoA derivatives.
The reaction mixture, which contained recombinant protein (125 ng/ml PGN_0725, 1.25 μg/ml PGN_1341, or 125 ng/ml PGN_1888), 200 mM sodium acetate, and 1 mM CoA derivatives, were incubated for 5 min. The concentration of CoA as a byproduct in the reaction mixture were measured. Data represent the mean ± standard deviations (n = 3).

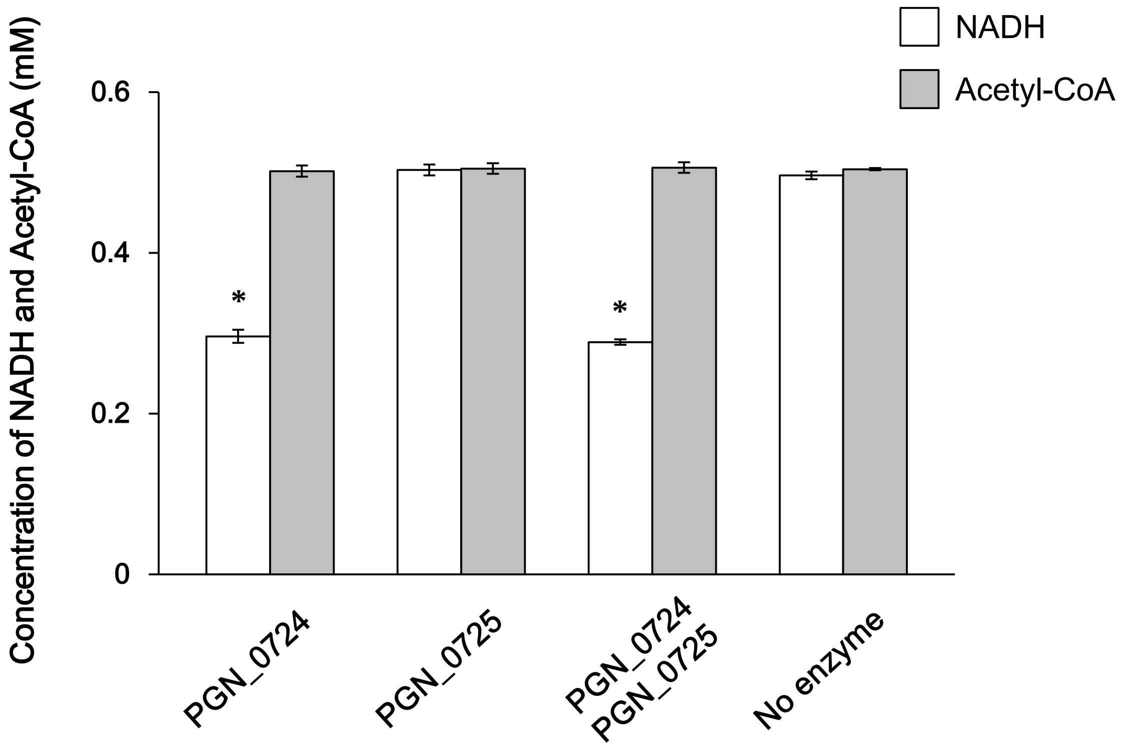

Figure S3. Consumption of NADH and acetyl-CoA by recombinant PGN_0724 and/or PGN_0725 proteins. Reaction mixtures containing 1.0 μg/mL recombinant protein, 0.5 mM NADH, 0.5 mM succinate semialdehyde, and 0.5 mM acetyl-CoA were incubated for 60 min. Concentrations of remaining NADH and acetyl-CoA in the reaction mixtures were measured. Data represent the mean ± standard deviation from three independent experiments. Asterisks indicate significant differences compared with the no-enzyme reaction mixture ($P < 0.01$).
